# Intravital microscopy of tumor vessel morphology and function using a standard fluorescence microscope

**DOI:** 10.1007/s00259-021-05243-0

**Published:** 2021-02-19

**Authors:** Jon-Vidar Gaustad, Trude G. Simonsen, Lise Mari K. Hansem, Einar K. Rofstad

**Affiliations:** Group of Radiation Biology and Tumor Physiology, Department of Radiation Biology, Institute for Cancer Research, Oslo University Hospital, Box 4953 Nydalen, 0424 Oslo, Norway

**Keywords:** Intravital microscopy, Window chamber, Vascular morphology, Vascular function, Tumor-associated lymphatics, Hypoxia

## Abstract

**Purpose:**

The purpose of the study was to demonstrate the performance and possible applications of an intravital microscopy assay using a standard fluorescence microscope.

**Methods:**

Melanoma and pancreatic ductal adenocarcinoma xenografts were initiated in dorsal window chambers and subjected to repeated intravital microscopy. The entire tumor vasculature as well as the normal tissue surrounding the tumor was imaged simultaneously with high spatial and temporal resolution. Vascular morphology images were recorded by using transillumination, and vascular masks were produced to quantify vessel density, vessel diameter, vessel segment length, and vessel tortuosity. First-pass imaging movies were recorded after an intervenous injection of a fluorescent marker and were used to investigate vascular function. Lymphatics were visualized by intradermal injections of a fluorescent marker.

**Results:**

The intravital microscopy assay was used to study tumor growth and vascularization, tumor vessel morphology and function, tumor-associated lymphatics, and vascular effects of acute cyclic hypoxia and antiangiogenic treatment. The assay was sensitive to tumor-line differences in vascular morphology and function and detected tumor-induced lymphatic dilation. Acute cyclic hypoxia induced angiogenesis and increased the density of small diameter vessels and blood supply times, whereas antiangiogenic treatment selectively removed small-diameter vessels, reduced blood supply times, and induced hypoxia. Moreover, the window chamber was compatible with magnetic resonance imaging (MRI), and parametric images derived by dynamic contrast-enhanced MRI were shown to reflect vascular morphology and function.

**Conclusions:**

The presented assay represents a useful and affordable alternative to intravital microscopy assays using confocal and multi-photon microscopes.

**Supplementary Information:**

The online version contains supplementary material available at 10.1007/s00259-021-05243-0.

## Introduction

The vasculature in normal tissues is strictly organized to secure sufficient supply of oxygen and nutrients and effective removal of waste products. In contrast, the vasculature in malignant tissue is highly abnormal and appears strikingly disorganized [1]. Individual tumor vessels are elongated and tortuous, and may show aberrant vessel diameters and vessel wall abnormalities (i.e. discontinuous endothelial lining, and lack of basement membrane and pericyte coverage) [2, 3]. Moreover, the vascular networks in most tumors display limited arterial supply, loss of vessel hierarchy, and highly heterogeneous vessel density [2–4]. These morphological abnormalities collectively increase the geometric resistance to blood flow, and, consequently, many tumors show unstable blood flow and low and heterogeneous blood supply [5–7]. The abnormal tumor vasculature plays a key role in the development of the hostile tumor microenvironment which is characterized by hypoxia, elevated interstitial fluid pressure, and extracelluar acidose [3, 8]. This hostile microenvironment has been shown to cause resistance to several treatment modalities, and has been demonstrated to induce malignant progression, invasive growth, and metastatic spread [8, 9].

Detailed studies of tumor vasculature have been performed by the use of window chamber preparations and intravital microscopy techniques [10–13]. These methods are particularly attractive because they allow high-resolution imaging of vascular morphology and function, and because the imaging can be repeated during growth and during treatments. In most studies, advanced microscopes such as confocal and multi-photon microscopes have been used [10, 11]. These imaging platforms provide excellent images, but the image acquisition and analysis are technically cumbersome and the microscopes are expensive and only available in specialized laboratories. Our laboratory has developed a dorsal window chamber compatible with magnetic resonance imaging (MRI), and an intravital microscopy assay for studying tumors growing in dorsal window chambers as well as the normal tissue surrounding the tumors. Importantly, this assay can be performed with a standard fluorescence microscope available in most laboratories. In the current communication, we present the intravital microscopy assay and describe experiments where we have used the assay to study tumor growth, vascularization, vessel morphology and function, lymphatics, and vascular effects of acute cyclic hypoxia and antiangiogenic treatment. Finally, we report an MRI-experiment demonstrating the potential of the MR-compatible window chamber for dynamic contrast-enhanced MRI (DCE-MRI).

## Methods

### Window chamber preparation

**Fig. 1a:**
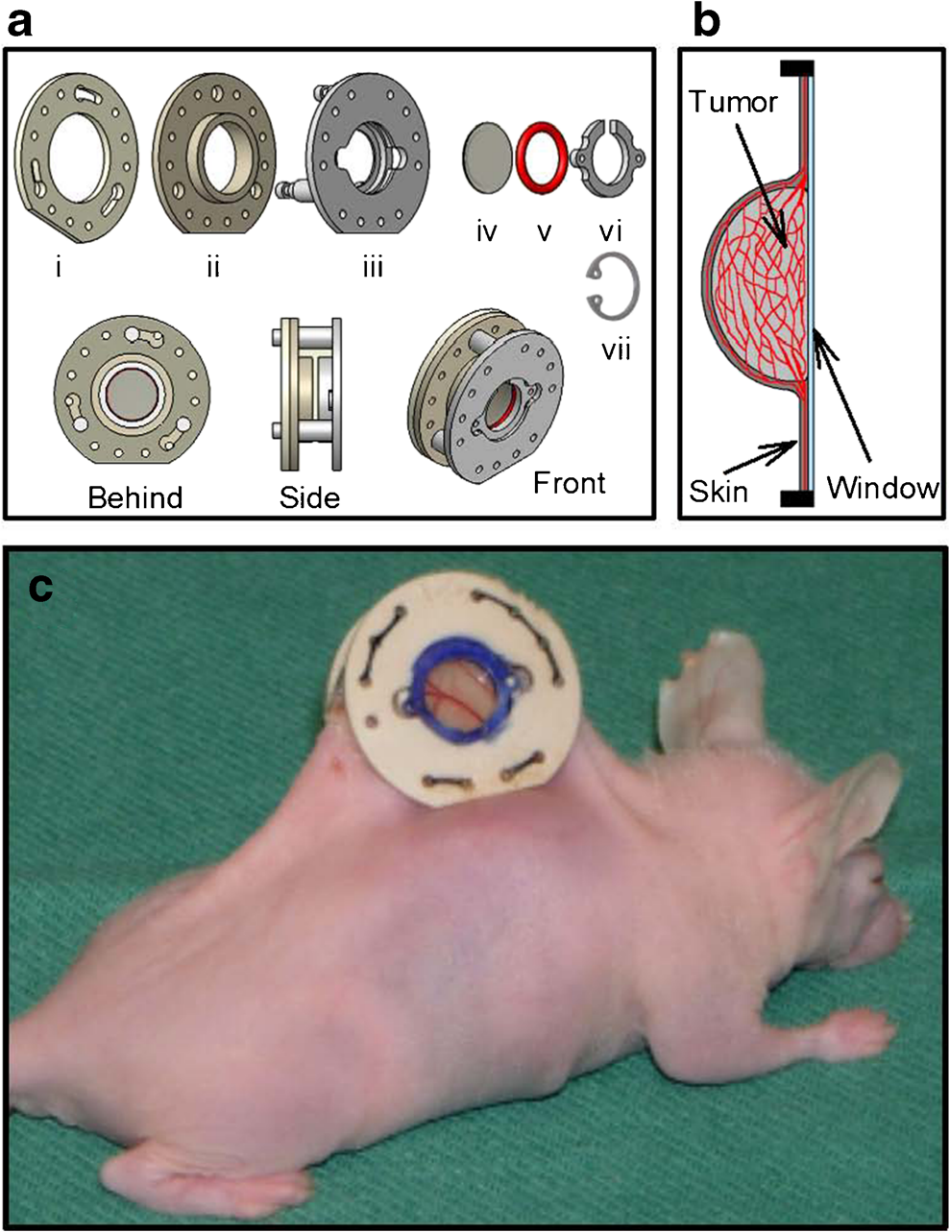
Technical drawing of our in-house made window chamber consisting of three frames (i–iii), a transparent plastic window with a diameter of 6.0 mm (iv), a silicon ring (v), and a plastic clip (vi). The window chamber is made of polymer material and has an outer diameter of 19.0 mm, an outer thickness of 7.0 mm, and a total weight of 1.0 g. In experiments that did not involve magnetic resonance imaging, the plastic clip (vi) was replaced by a c-ring in stainless steel (vii). **b** A schematic illustration of a transversal section through a tumor growing in the window chamber. The tumor (light gray color) grows as a hemisphere surrounded by the plastic window (light blue color) and normal skin (dark gray color). The vasculature (red color) in the tumor and the surrounding normal skin can be studied by intravital microscopy through the transparent plastic window. **c** Photograph of the window chamber implanted in the dorsal skin fold of a BALB/c *nu/nu* mouse. Modified from Gaustad et al. and Simonsen et al. [14, 44]

A technical drawing of our in-house-made window chamber, a schematic illustration of a tumor growing in the window chamber, and a photograph of the window chamber implanted in the dorsal skin of a BALB/c *nu/nu* mouse are shown in Fig. 1. The surgical implantation procedure has been described in detail previously and was performed on anesthetized mice [14]. Briefly the window chamber consisted of parallel frames that sandwiched an extended double layer of skin. Before the chamber was implanted, a circular hole was made in one of the skin layers. A plastic window was attached to the frame on the surgical side and provided visual access to the fascial side of the opposite skin layer. Tumors were initiated by implanting multicellular spheroids or solid tumor pieces with a diameter of 100 to 400 μm onto the fascial side of the intact skin layer, and grew as hemispheres surrounded by the plastic window and normal skin (Fig. 1b).

### Intravital microscopy imaging

We used a standard inverted fluorescence microscope (IX-71; Olympus, Munich, Germany) and a black and white CCD camera (C9300–024; Hamamatsu Photonics, Hamamatsu, Japan) to study tumors growing in window chambers. Tumor vasculature was visualized by using transillumination and filters for green light, or fluorescence imaging after a 0.2 mL bolus of tetramethylrhodamine isothiocyanate-labeled dextran (TRITC-dextran; molecular weight 155 kDA; Sigma-Aldrich, Scnelldorf, Germany) was injected into the lateral tail vein as detailed previously [14, 15]. First-pass imaging movies were recorded at a rate of 22.3 frames per second by using a × 2 objective lens, resulting in a time resolution of 44.8 ms, a field of view of 6.0 × 6.0 mm^2^, and a pixel size of 7.5 × 7.5 μm^2^, whereas high-resolution images of the tumor vasculature were recorded by using a × 4 objective lens, resulting in a field of view of 3.8 × 3.8 mm^2^ and a pixel size of 3.7 × 3.7 μm^2^. Lymphatics were visualized by 3–5 intradermal injections of ~2 μl TRITC-dextran in the normal skin tissue surrounding the window chambers. Mice with window chambers were anesthetized and the window chambers were screwed to the microscope stage during intravital microscopy to avoid movement caused by respiration, and the body core temperature was kept at 37–38 °C by using a hot-air generator.

### Immunohistochemical detection of tumor hypoxia

The tumors were resected immediately after the last intravital microscopy examination and fixed in phosphate-buffered 4% paraformaldehyde. Pimonidazole [1-[(2-hydroxy-3-piperidinyl)-propyl]-2-nitroimidazole], administered as described previously [15], was used as a hypoxia marker. An anti-pimonidazole rabbit polyclonal antibody (Professor James A. Raleigh, University of North Carolina, Chapel Hill, NC, USA) was used as primary antibody. Diaminobenzidine was used as chromogen, and hematoxylin was used for counterstaining. Hypoxic fractions were assessed by image analysis and were defined as the area fraction of the viable tissue showing positive pimonidazole staining.

### DCE-MRI

Dynamic contrast-enhanced magnetic resonance imaging (DCE-MRI) was performed by using a 1.5 T whole-body scanner (Signa; General Electric, Milwaukee, WI) and a cylindrical slotted tube resonator transceiver coil especially constructed for mice [16]. Gd-DTPA (Schering, Berlin, Germany), diluted to a final concentration of 0.06 M was used as contrast agent and was administered in the tail vein of the mice in a bolus dose of 5.0 ml/kg. Two calibration tubes, one with 0.5 mM Gd-DTPA in 0.9% saline and the other with 0.9% saline only, were placed adjacent to the mice in the coil. The tumors were imaged sagittally in a single scan adjacent to and parallel to the window of the chamber preparations at a voxel size of 310 × 310 × 2000 μm^3^. T_1_-weighted images (TR = 200 ms, TE = 3.2 ms, and α = 80°) were recorded at a time resolution of 14 s. Two proton density images (TR = 900 ms, TE = 3.2 ms, and α = 20°) and three T_1_-weighted images were acquired before Gd-DTPA was administered, and T_1_-weighted images were recorded for 15 min after the administration of Gd-DTPA. Gd-DTPA concentrations were calculated from signal intensities by using the method described by Hittmair [17], and parametric images of *K*^trans^ were generated from concentration versus time series by using the arterial input function of Benjaminsen et al. [18] and Tofts generalized pharmacokinetic model [19].

### Hypoxia treatment

Unanesthetized mice were placed in an in-house-made incubation chamber and exposed to a continuous flow of a humidified gas mixture at room temperature to induce hypoxia. The hypoxia treatment consisted of 12 cycles of 10 min of 8% O_2_ in N_2_ followed by 10 min of air for a total of 4 h. Control mice were exposed to a continuous flow of humidified air for 4 h. The hypoxia treatment began on the first day after tumor initiation and was given once per day for 9 days. 8 tumor-bearing mice were included in the treatment and control groups.

### Sunitinib treatment

Mice were divided in groups with matched tumor size, and were treated with 40 mg/kg/day sunitinib (treatment group, 8 mice) or vehicle (control group, 6 mice) for 4 days. The treatment started 12 days after tumor initiation, and at that time the tumors had developed vascular networks. Sunitinib and vehicle were administered orally by gavage. Sunitinib L-malate (LC Laboratories, Woburn, MA) was dissolved in hydrochloric acid (1.0 M ratio of sunitinib), polysorbate 80 (0.5%; Sigma-Aldrich, Schnelldorf, Germany), polyethylene glycol 300 (10%; Sigma-Aldrich), sodium hydroxide (to adjust pH to 3.5), and sterile water.

### Statistical analysis

Statistical comparisons of data were carried out by using the Student’s t test (single comparisons) or by one-way analysis of variance followed by the Student–Neuman–Keuls test (multiple comparisons) when the data complied with the conditions of normality and equal variance. Under other conditions, comparisons were carried out by non-parametric analysis using the Mann–Whitney rank-sum test (single comparisons) or the Kruskal–Wallis analysis of variance on ranks test followed by the Dunn’s test (multiple comparisons). The Pearson product moment correlation test was used to search for correlations between two parameters. Probability values of *p* < 0.05, determined from two-sided tests, were considered significant. The statistical analysis was performed by using the SigmaStat statistical software (SPSS Science, Chicago, IL, USA).

## Results

### Monitoring tumor growth and vascularization

**Fig. 2a:**
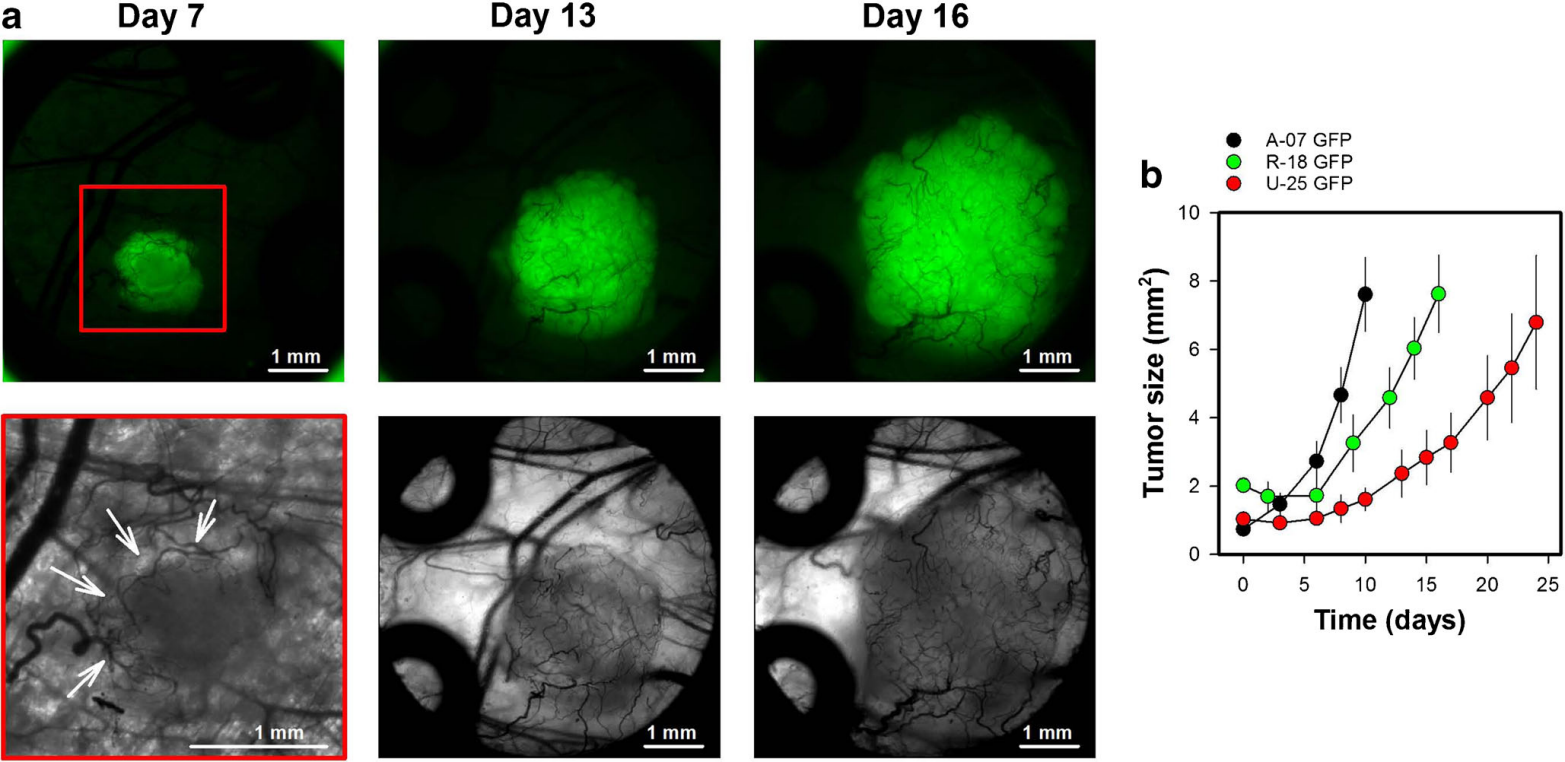
Intravital microsopy images of a R-18-GFP melanoma xenograft recorded 7, 13, and 16 days after tumor initiation. The upper row shows fluorescence images of the GFP-expressing tumor cells, and the lower row shows transillumination images of vessels within the tumor mass and in the tissue surrounding the tumor. A red box highlight the area shown in the transillumination image recorded on day 7 and white arrows highlight novel tumor vessels in the tumor periphery. The fluorescence and transillumination images recorded on day 13 and 16 show the same field of view. **b** Tumor size versus time for A-07-GFP, R-18-GFP, and U-25-GFP melanoma xenografts. Tumor size was depicted from the area showing positive GFP signal. Points, means of 6–13 tumors; bars, standard error. Fig. 2b was modified from Gaustad et al. [15]

The implanted window chambers allowed repeated intravital microscopy, and were imaged thrice a week to study the growth and the initial vascularization of tumors. Figure 2a shows intravital microscopy images of a representative R-18-GFP melanoma xenograft, and illustrates the first signs of angiogenesis (day 7; novel vessels in the tumor periphery), and the vascular network after the entire tumor was vascularized (day 13 and 16). By using tumor cells transfected with green fluorescent protein (GFP), the tumor cells were easily identified in fluorescence images and the tumor size was assessed by measuring the area showing positive GFP-signal. Fig. 2b shows plots of tumor size versus time for three melanoma xenograft models. The melanoma models differed in growth rate (*p* < 0.05), and these differences reflected differences in the angiogenic activity. Thus A-07-GFP tumors produced a vessel length of 13.5 mm/day and showed the highest growth rate, R-18-GFP tumors produced a vessel length of 7.5 mm/day and showed the second highest growth rate, and U-25-GFP tumors produced a vessel length of 4.1 mm/day and showed the lowest growth rate. We have studied tumors growing in window chambers for time periods up to four weeks. This period is limited by the time the tumors need to outgrow the window chamber.

**Fig. 3a:**
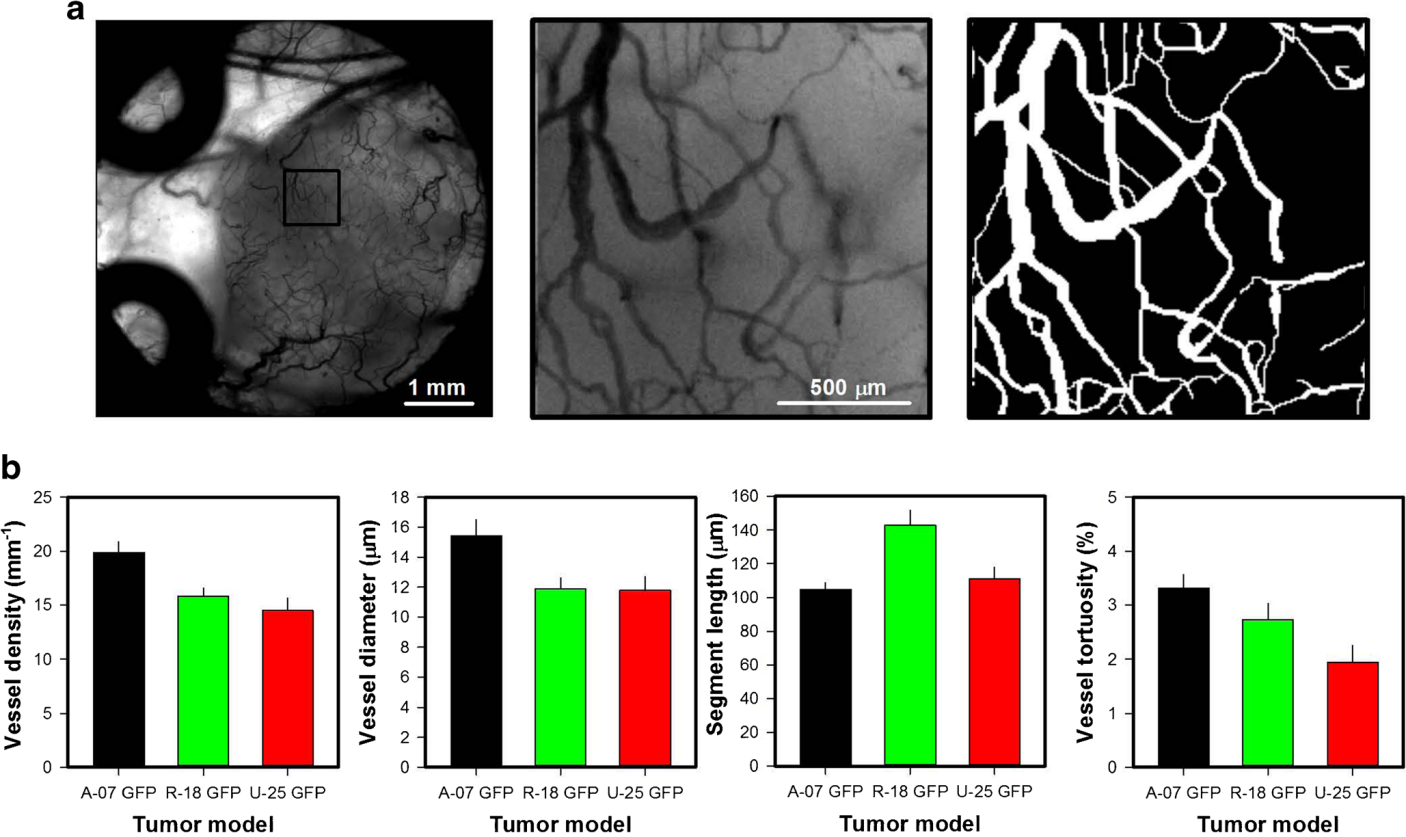
Low and high-magnification intravital microscopy images and high-magnification image of the vascular mask of a R-18-GFP melanoma xenograft. **b** Vessel density, vessel diameter, vessel segment length, and vessel tortuosity in A-07-GFP, R-18-GFP, and U-25-GFP melanoma xenografts. Points, means of 6–13 tumors; bars, standard error. Fig. 3b was modified from Gaustad et al. [15]

**Fig. 4a:**
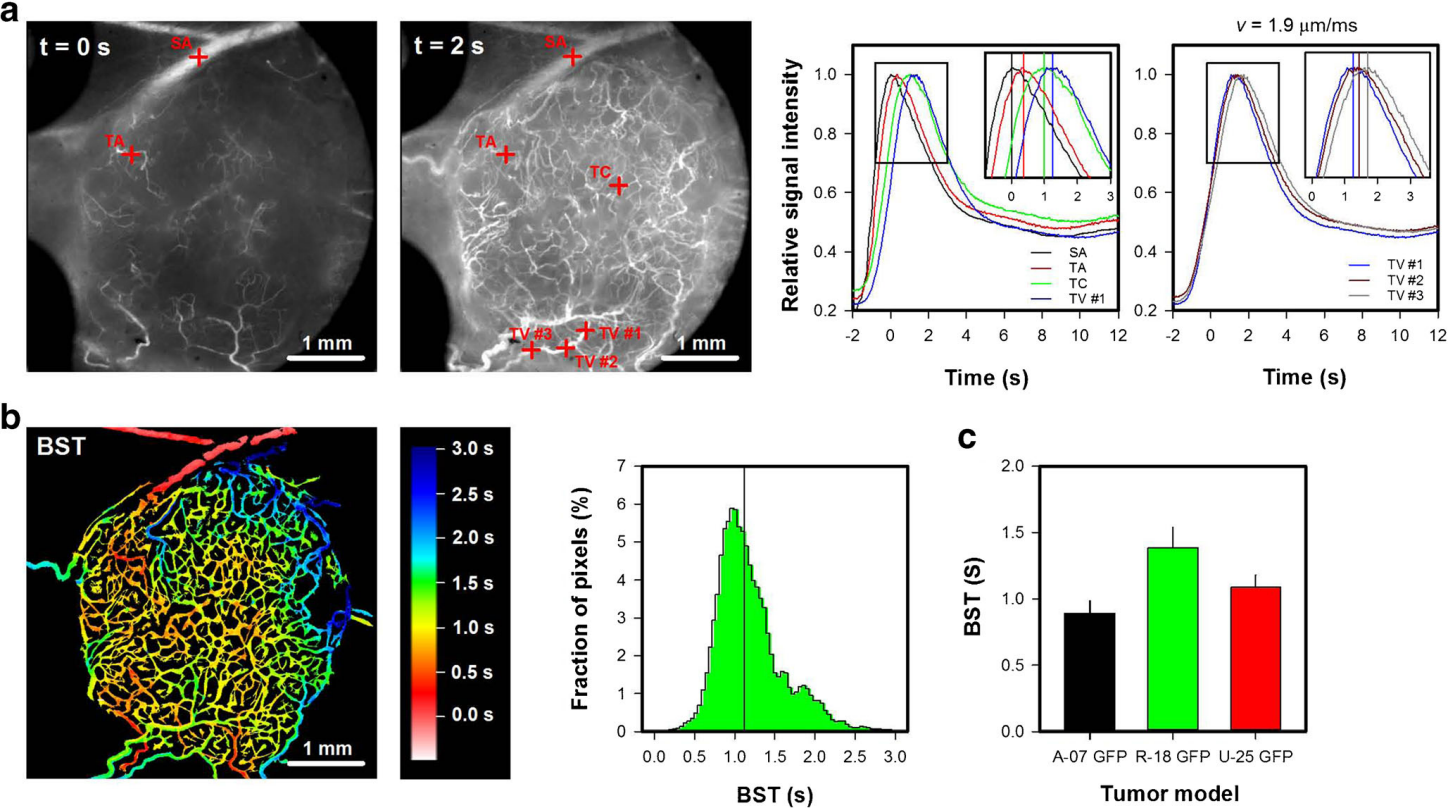
Single frames from a first-pass imaging movie of a R18-GFP melanoma xenograft, and relative signal intensity versus time for the vessels marked in the frames. The entire first-pass imaging movie can be seen in Online Resource 1. The signal intensity curves refer to the main supplying artery (SA; black curve), a tumor arteriole (TA; red curve), a tumor capillary (TC; green curve), and a tumor venule (TV #1–3; blue, dark red, and gray curve). In the tumor venule, three regions of interest were selected. The delay in the signal intensity curves recorded along the tumor venule was used to calculate the blood flow velocity, which was 1.9 μm/ms. **b** Color-coded blood supply time image (BST) image and BST histogram of the R-18-GFP tumor. BST refer to the time arterial blood needs to flow from the main supplying artery to a tumor vessel, and was calculated from the delay in signal intensity curves for every vessel pixel. The BST scale is given by the color bar. The vertical line in the BST histogram shows median BST. **c** BST in A-07-GFP, R-18-GFP, and U-25-GFP melanoma xenografts. Columns, means of 7–17 tumors; bars, standard error. Fig. 4c was modified from Gaustad et al. [15]

**Fig. 5a:**
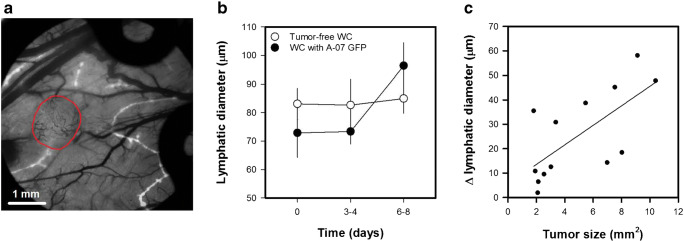
Intravital microscopy image of TRITC-dextran filled lymphatics (white) superimposed on transillumination image of blood vessels (black). The image shows an A-07-GFP melanoma xenograft (delineated by red line) and the surrounding normal tissue. Images of lymphatics were recorded 10–15 min after 3–5 intradermal injections of ~2 μl TRITC-dextran in the skin surrounding the window chambers. **b** Lymphatic diameter versus time for window chambers with and without A-07-GFP tumors. Points, means of 8–13 window chambers; bars, standard error. **c** Change in lymphatic diameter versus tumor size. Points, individual window chambers; line, curve fitted to the data by linear regression. Fig. 5b-c was modified from Gaustad et al. [45]

**Fig. 6a:**
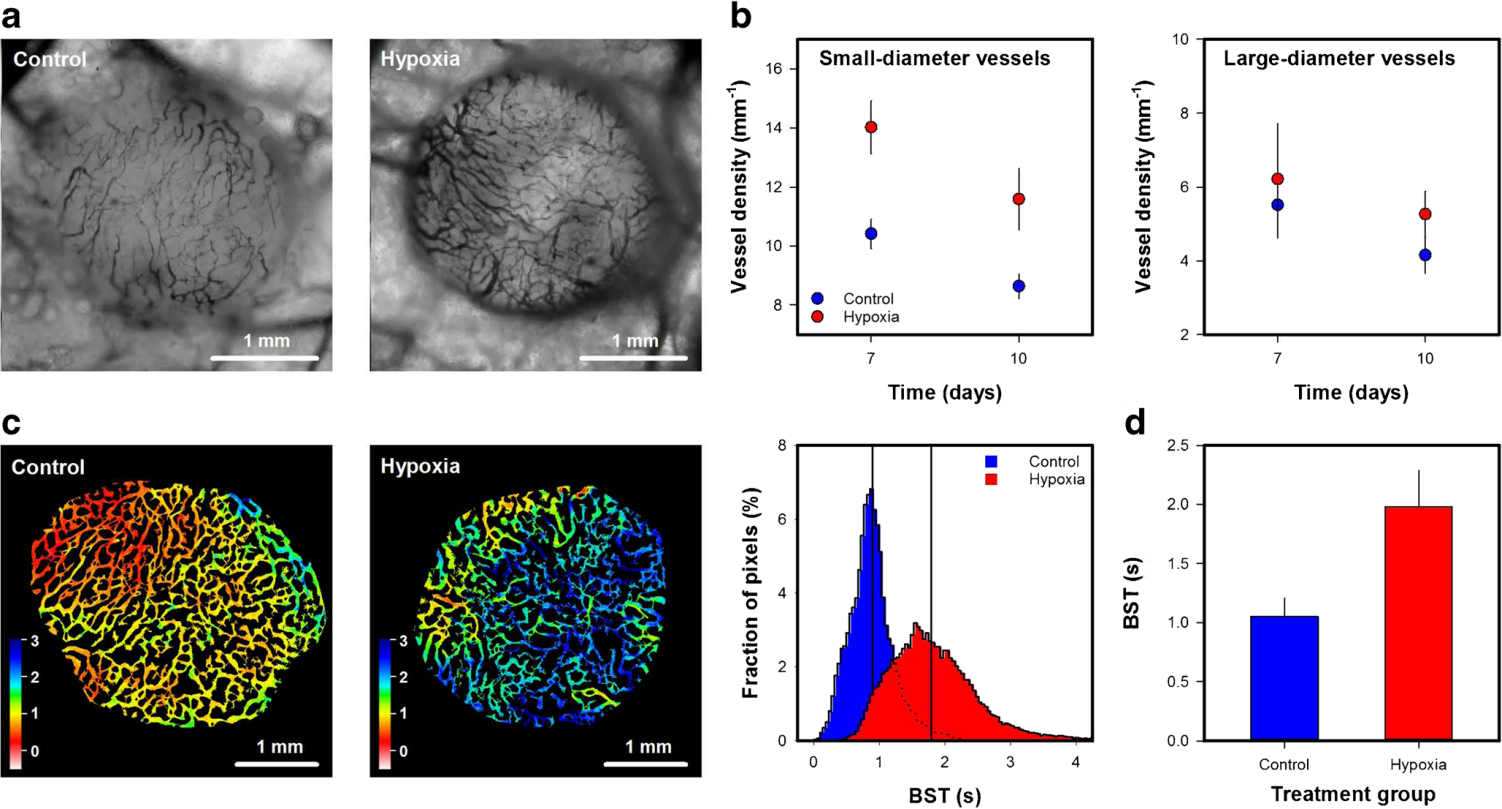
Intravital microscopy images of an untreated (control) and a hypoxia-treated A-07-GFP melanoma xenograft. The hypoxia treatment consisted of 12 cycles of 10 min of low O_2_ (8%) followed by 10 min of air. The treatment started the first day after the tumors were implanted in window chambers, and was given daily for 9 days. **b** Density of small-diameter (< 20 μm) and large-diameter vessels (> 20 μm) in untreated and hypoxia-treated A-07-GFP tumors. **c** Blood supply time (BST) image and BST histogram of an untreated and a hypoxia-treated A-07-GFP tumor. The BST scale is given by the color bar. The vertical lines in the BST histograms show median BST. **d** BST in untreated and hypoxia-treated tumors. Points and columns, means of 6–8 tumors; bars, standard error (**b** and **d**). Fig. 6b and d was modified from Gaustad et al. [46]

**Fig. 7a:**
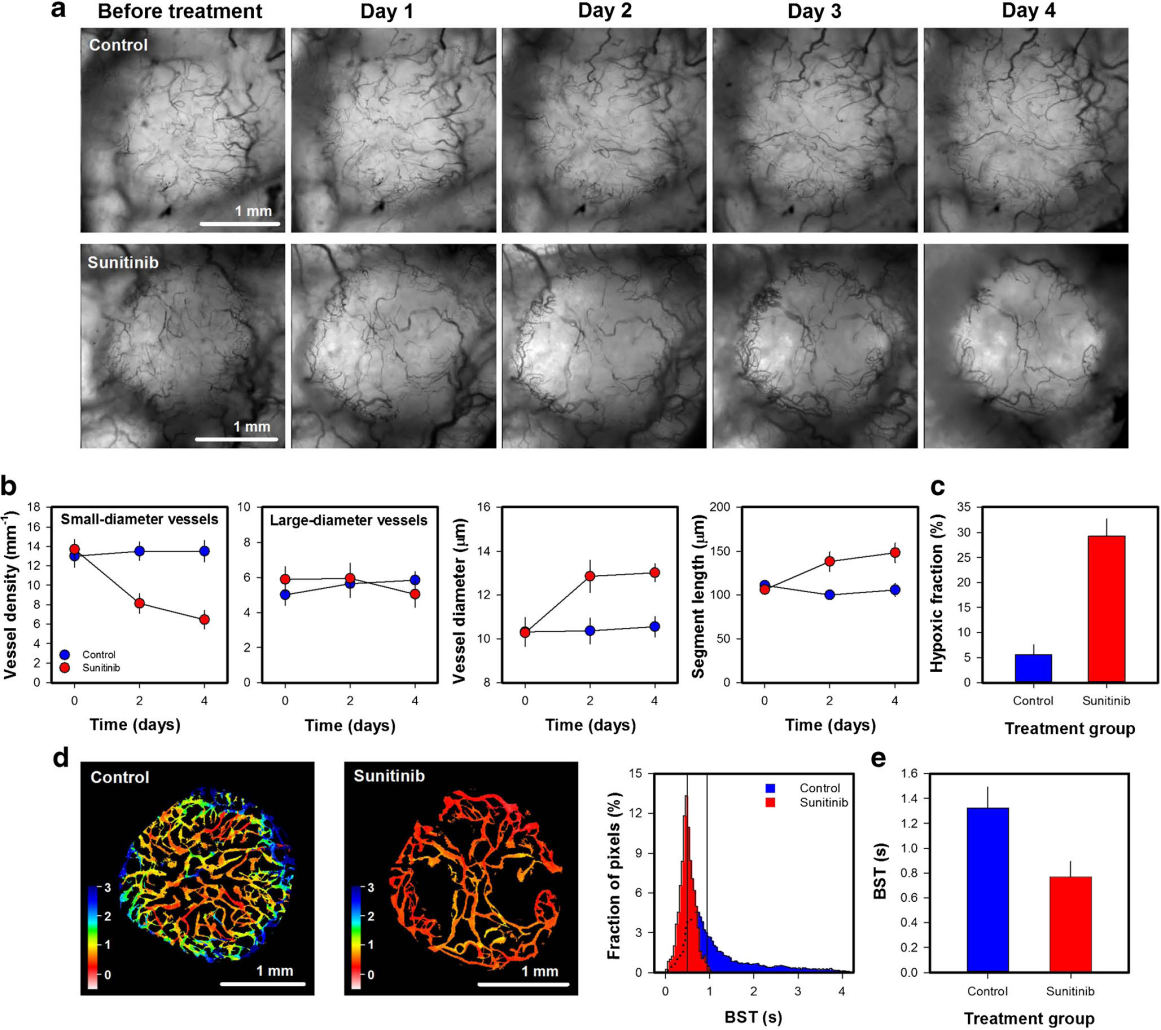
Intravital microscopy images of an untreated (control) and a sunitinib-treated Capan-2 pancreatic ductal adenocarcinoma xenograft. The tumor-bearing mice were given daily doses of 40 mg/kg sunitinib or vehicle for 4 days. **b** Density of small-diameter vessels (< 10 μm), density of large-diameter vessels (> 10 μm), vessel diameter, and vessel segment length versus time in untreated and sunitinib-treated Capan-2 tumors. **c** Hypoxic fraction in untreated and sunitinib-treated Capan-2 tumors. The hypoxic fractions were determined by immuohistochemistry using pimonidazole as a hypoxia marker. **d** Blood supply time (BST) image and BST histogram of an untreated and a sunitinib-treated Capan-2 tumor. The BST scale is given by the color bar. The vertical lines in the BST histograms show median BST. **e** BST in untreated and sunitinib-treated tumors. Points and columns, means of 6–8 tumors; bars, standard error (**b-c** and **e**). Fig. 6b-c and e was modified from Gaustad et al. [47]

**Fig. 8a:**
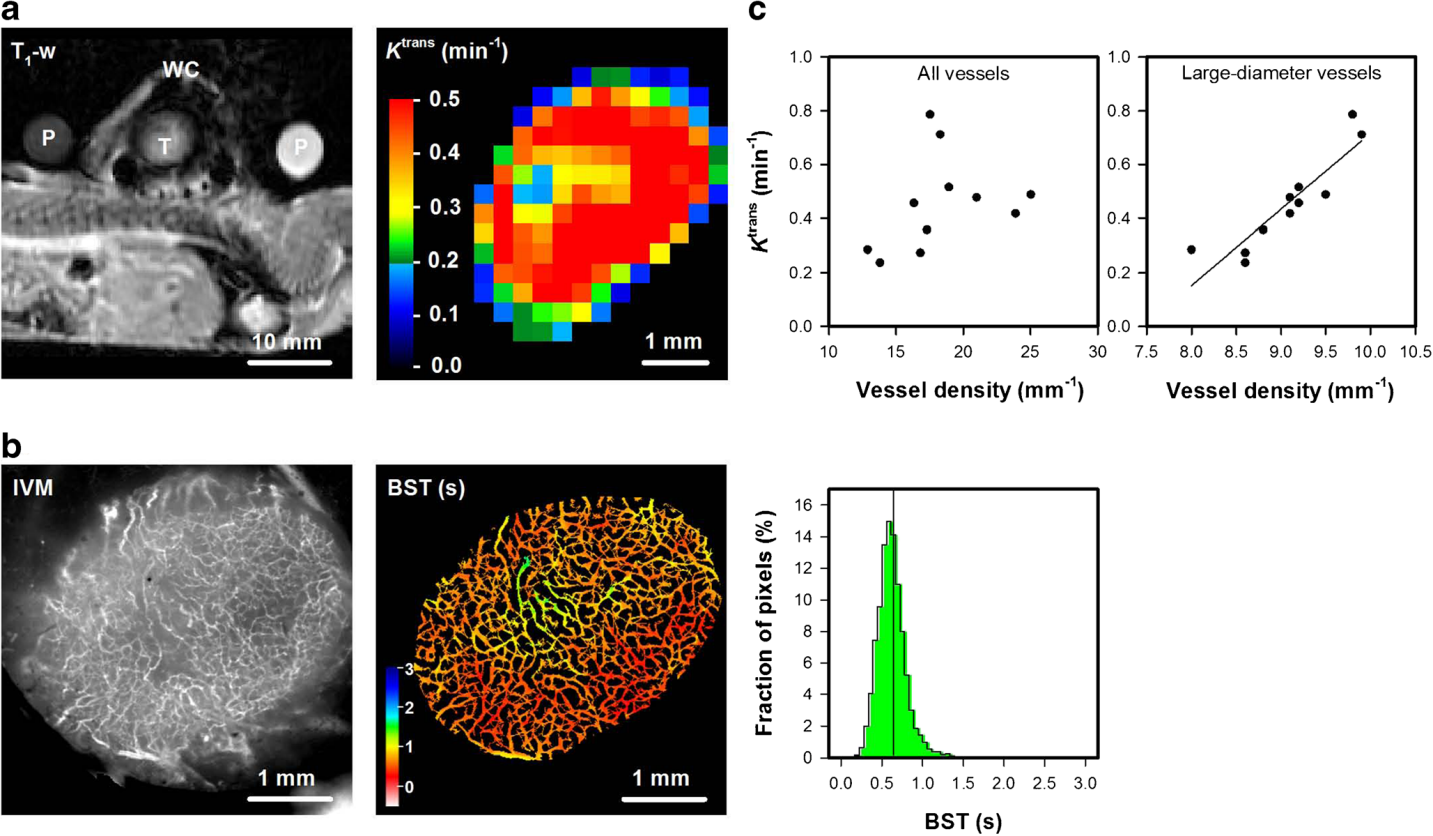
A T_1_-weighted magnetic resonance (MR) image of calibration phantoms (P) and a mouse bearing a window chamber (WC) with an A07-GFP melanoma xenograft (T), and a color-coded *K*^trans^ image of the A07-GFP tumor calculated from dynamic contrast-enhanced MR data by using Tofts generalized pharmacokinetic model. The *K*^trans^ scale is given by the color bar. **b** Intravital microscopy image of TRITC-dextran filled tumor vessels and blood supply time (BST) image of the A-07-GFP tumor. The BST scales are given by the color bar. **c** Median *K*^trans^ versus the density of all vessels and median *K*^trans^ versus the density of large-diameter vessels (> 20 μm) in A-07-GFP tumors. Points, individual tumors. Fig. 8c was modified from Gaustad et al. [14]

### Quantitative studies of vascular morphology and function

Figure 3a shows intravital microscopy images and the vascular mask of a representative R-18-GFP melanoma xenograft. The microscopy images were recorded by using transillumination and a filter for green light, and visualize vessels perfused with red blood cells because these cells absorb green light. Importantly, the contrast between blood vessels and tissue in the images was sufficient to create accurate vascular masks. The vascular masks were used to calculate morphological parameters describing the tumor vasculature including vessel length densities (i.e., total vessel length per mm^2^ tumor area) and vessel diameters. Interestingly, the three melanoma xenograft models differed substantially in vascular morphology (Fig. 3b). Thus, A-07-GFP tumors showed higher vessel density and larger vessel diameters than R-18-GFP and U-25-GFP tumors (*p* < 0.05), R-18-GFP tumors showed longer vessel segments than A-07-GFP and U-25-GFP tumors (*p* < 0.05), and A-07-GFP tumors showed more tortuous vessels than U-25-GFP tumors (*p* < 0.05).

To study vascular function, first-pass imaging movies were recorded. Different vessel types could be identified in the first-pass imaging movies. Tumor arterioles were supplied shortly after the main supplying artery, tumor venules were supplied last and showed high vessel diameters, and tumor capillaries were supplied after the tumor arterioles and before the tumor venules and showed low vessel diameters. A first-pass imaging movie, illustrating how a bolus of TRITC-dextran moves through the vascular network of a representative R-18-GFP tumor and the surrounding normal tissue, is presented in Online Resource 1. Figure 4a shows single frames from the movie and plots of relative signal intensity versus time for the vessels marked in the frames. The first frame was recorded when the bolus reached the main supplying artery, and the second frame was recorded 2 s later when the bolus had reached most tumor vessels. The peak in the signal intensity curve appeared first in the main supplying artery (SA; black curve) and was delayed in the selected tumor arteriole (TA; red curve), tumor capillary (TC; green curve), and tumor venule (TV #1, blue curve). The blood supply time (BST) was determined by measuring the time delay, and thus described the time needed for arterial blood to flow from the main supplying artery to a tumor vessel. A color-coded BST image and a BST histogram of the representative R-18-GFP tumor are shown in Fig. 4b. The sensitivity of the BST measurements is illustrated by the signal intensity curves. Thus, the curves depicted in different tumor vessels (TA, TC, and TV) could easily be separated, and gradients along vessel segments (TV #1–3) could be detected. Gradients along vessel segments were used to calculate the blood flow velocity. The blood flow velocity in the selected TV was 1.9 μm/ms, which is well within the range of tumor blood flow velocities reported by others [6, 11]. R-18-GFP tumors showed higher BST values and thus lower blood flow velocities than A-07 GFP tumors (Fig. 4c; *p* < 0.01), whereas significant differences in BST were not found between A-07-GFP and U-25GFP tumors or between R-18-GFP and U-25-GFP tumors (Fig. 4c; *p* > 0.05).

### Imaging lymphatics

Lymphatics were visualized by multiple intradermal injections of ~2 μl TRITC-dextran in the normal skin tissue surrounding the window chambers (Fig. 5a), and the imaging protocol was repeated for window chambers with and without A-07-GFP melanoma xenografts. The diameter of adjacent lymphatics was increased 6–8 days after tumor implantation (Fig. 5b; *p* < 0.001), whereas the lymphatic diameters did not change in tumor-free window chambers (Fig. 5b; *p* > 0.05). Moreover, a positive correlation was found between the increase in lymphatic diameter and tumor size (Fig. 5c; *p* < 0.05; *R*^2^ = 0.46), implying that the A-07-GFP tumors induced lymphatic dilation. Others have shown that tumor cells influence lymphatics by secreting vascular endothelial growth factor C (VEGF C), and increases in the diameter of adjacent lymphatics have been observed after implantation of VEGF-C overexpressing fibrosarcoma xenografts in the tip of the mouse ear [20–22]. Importantly the increase in lymphatic diameters was demonstrated to increase the propensity for lymph node metastasis, and could be inhibited by blocking the VEGF-C pathway [21, 22].

### Vascular effects of acute cyclic hypoxia

To investigate vascular effects of acute cyclic hypoxia, tumor-bearing mice were periodically exposed to a low oxygen atmosphere. Figure 6a shows intravital microscopy images of an untreated and a hypoxia-treated A-07-GFP melanoma xenograft. Quantitative studies revealed increased density of small-diameter vessels (< 20 μm) in hypoxia-treated tumors (Fig. 6b; *p* < 0.05), whereas the density of large-diameter vessels (> 20 μm) did not differ between untreated and hypoxia treated tumors (Fig. 6b; *p* > 0.05). These observations imply that the hypoxia-treatment induced angiogenesis in A-07-GFP window chamber tumors, and corresponds well to a previous study of intradermal A-07 tumors. In the previous study, hypoxia-treatment induced angiogenesis and increased the propensity for pulmonary metastasis by increasing the expression of VEGF-A [23]. Fig. 6c shows the BST image and the BST histogram of an untreated and a hypoxia treated A-07-GFP tumor. The hypoxia-treated tumors showed higher BST values and thus lower blood flow velocities than untreated tumors (Fig. 6d; *p* < 0.05). The difference in vascular function could most likely be attributed to differences in the geometric resistance to blood flow. When laminar flow can be assumed, the geometric resistance in a single vessel is inversely proportional to the vessel diameter in the fourth power [6]. Small-diameter vessels are thus expected to have high geometric resistance to blood flow, and if a substantial number of vessels become narrower or are replaced by narrower vessels, the geometric resistance in vascular networks can increase.

### Vascular effects of antiangiogenic treatment

Sunitinib inhibits the VEGF-A pathway by blocking the VEGF receptors [24]. To investigate vascular effects of sunitinib treatment, untreated and sunitinib-treated Capan-2 pancreatic ductal adencarcinoma (PDAC) xenografts were subjected to daily intravital microscopy (Fig. 7a). Sunitinib-treated tumors showed reduced density of small-diameter vessels (< 10 μm; Fig. 7b; *p* < 0.01), but did not differ from untreated tumors in the density of large-diameter vessels (> 10 μm; Fig. 7b; *p* > 0.05). The removal of small-diameter vessels resulted in increased vessel diameter and segment length (Fig. 7b; *p* < 0.01), and increased hypoxic fraction as revealed by immunohistochemistry using pimonidazole as a hypoxia marker (Fig. 7c; *p* < 0.01). Moreover, sunitinib-treated tumors showed lower BST values than untreated tumors. This is illustrated qualitatively in Fig. 7d which shows the BST image and the BST histogram of representative untreated and sunitinib-treated tumors, and quantitatively in Fig. 7e which shows BST of all the included tumors (*p* < 0.05). The reduced BST values could probably be attributed to reduced geometric resistance to blood flow. As discussed above, small-diameter vessels are expected to have a high geometric resistance to blood flow, and a selective removal of small-diameter vessels can thus reduce the geometric resistance in vascular networks. Improved vascular function after antiangiogenic treatment has also been reported by others [25, 26]. The phenomenon has been termed vascular normalization and has been shown to increase tumor oxygenation in some but not all tumor models [26–30]. To increase tumor oxygenation, the improved vascular function must outweigh the loss of tumor vessels [26]. In the experiment reported here, sunitinib reduced BST implying that the treatment increased blood flow velocity. Increased blood flow velocity can increase oxygen supply (i.e. increase the flow of oxygen-carrying erythrocytes), but this effect was probably too small to compensate for the loss of tumor vessels as the treatment increased hypoxic fractions (Fig. 7c). It has also been argued that increased blood flow velocity can decrease microvascular transit times which limit the time available for blood-tumor oxygen exchange [31, 32]. Interestingly, Østeergaard et al. demonstrated that reduced microvascular transit times can decrease tumor oxygenation despite increases in blood flow velocities by using mathematical simulations [33]. The sunitinib-induced reduction in BST reported here may thus have contributed to increased hypoxic fraction by decreasing the time available for blood-tumor oxygen exchange.

### Comparison/combination with MRI

The window chamber was made of polymer material and was thus compatible with MRI. Figure 8a shows a T_1_-weigthed MR image of a mouse with an A-07-GFP window chamber tumor, and a color-coded *K*^trans^ image of the tumor obtained by DCE-MRI. The MR images were recorded with an in-plane resolution of 310 × 310 μm^2^ and a slice thickness of 2000 μm, and, consequently, individual tumor vessels could not be distinguished in these images. By subjecting the same tumor to intravital microscopy, the *K*^trans^ image could be compared with morphological and functional images of the tumor vasculature (Fig. 8b; in-plane resolution 7.5 × 7.5 μm^2^). The A-07-GFP tumor did not display avascular regions and showed low BST values compared to most A-07-GFP, R-18-GFP, and U-25-GFP tumors (Fig. 4). The intravital microscopy images thus implied that the blood supply of the tumor was highly efficient, and accordingly the tumor showed high *K*^trans^ values. Moreover, positive correlations were found between *K*^trans^ and vessel density when all the A-07-GFP tumors were included (Fig. 8c). Interestingly, the strongest correlations were found between *K*^trans^ and the density of large-diameter vessels (> 20 μm; Fig. 8c; *p* < 0.001; *R*^2^ = 0.81), implying that *K*^trans^ was strongly influenced by these vessels.

## Discussion

The presented intravital microscopy assay used a standard fluorescence microscope to study normal tissue and tumor vasculature, as well as lymphatics in the skin surrounding tumors. Images of vascular morphology were recorded with high in-plane resolution to produce accurate vascular masks for calculating morphological parameters. Although the standard microscope provided excellent in-plane resolution, it did not provide depth resolution. The intravital microscopy images thus showed two-dimensional projections of the three-dimensional vasculature, and are expected to overestimate vessel densities because of contributions of vessels at different depths (from the window surface to a depth of ~1–2 mm determined by the penetration length of light). More advanced microscopes, such as confocal and multi-photon microscopes, provide three-dimensional resolution and do not share this limitation [10, 13]. However, it should be noticed that tumor-line differences as well as treatment-induced changes in vessel density were detected by using the 2-dimensional projections, implying that the assay provided important information on the relative vessel density. Moreover, when comparing intravital microscopy images with MRI-derived images, the depth resolution of the intravital microscopy images is not crucial because the MR-scans have a substantial thickness (2 mm in the current study). Calculations of vessel diameter, vessel segment length, and vessel tortuosity are not expected to be influenced by the lack of depth resolution.

An important advantage with our intravital microscopy assay is that the entire tumor vasculature and the surrounding normal tissue can be imaged simultaneously with high spatial and temporal resolution. To investigate vascular function, first-pass imaging movies were recorded with a temporal resolution of 44.8 ms, a spatial resolution of 7.5 × 7.5 μm^2^, and a field of view that covered the entire window chamber. These temporal and spatial resolutions were sufficient to identify the majority of the tumor vessels and to calculate accurate BST values for individual vessel pixels. With confocal and multi-photon microscopes, reduced temporal resolution or a smaller field of view must be accepted because scanning at multiple depths is highly time consuming [10, 13].

The intravital microscopy assay was used to investigate vascular effects of acute cyclic hypoxia and antiangiogenic treatment. As discussed above, the hypoxia-treatment induced angiogenesis by increasing VEGF-A expression, and the sunitinib treatment inhibited angiogenesis by targeting the VEGF receptors. Interestingly, the two treatments had opposite effects on vascular morphology and function. Thus the hypoxia treatment increased the number of small-diameter vessels and increased BST, whereas the sunitinib treatment selectively removed small-diameter vessels and reduced BST. The sunitinib treatment also induced hypoxia because the increased blood flow velocities were insufficient to compensate for the loss of tumor vessels and possibly because the reduced BST decreased the time available for blood-tumor oxygen exchange. These experiments demonstrate the strength of assessing both vascular morphology and function, and the feasibility of the intravital microscopy assay to provide such information.

Vascular networks in both normal and tumor tissues show vessel pathways with varying lengths, and if short pathways are enlarged, the blood flow may bypass long pathways and form functional shunts [34]. Functional shunts may deprive the regions that are supplied by the long pathways their supply of oxygen and nutrients, and are prevented in normal tissues by vascular communication and structural adaptation [35, 36]. In tumor tissues, functional shunts are frequently observed possibly because the vascular communication is impaired [37]. Pries et al. suggested that antiangiogenic treatment may restore the vascular communication and prevent functional shunts, and demonstrated that this may increase tumor oxygenation by using mathematical simulations [35]. Our intravital microscopy assay may be used to study the occurrence and consequences of functional shunts in tumor models. Vessel diameters and blood flow directions and velocities can be assessed in first-pass imaging movies to identify functional shunts, and the imaging may be repeated to investigate possible effects of antiangiogenic treatments.

Tumor cells transfected with GFP were used in the experiments reported here. The GFP-signal allowed accurate determination of tumor size and location, and was used to study tumor growth. GFP transfected tumor cells have also been used to study metastatic spread, and several live fluorescent reporters have been established to monitor gene expression and regulation by intravital microscopy [11, 13, 21]. Cao et al. genetically engineered tumor cells with GFP as a reporter for hypoxia inducible factor 1 (HIF-1) activation, and demonstrated that the intitial angiogenesis preceded HIF-1 activation [38]. Interestingly, the HIF-1 activation was monitored by intravital microscopy using a standard fluorescence microscope, suggesting that similar experiments can be performed with the intravital microscopy assay presented here. Intravital microscopy techniques for imaging the metabolic environment of tumors growing in window chambers have also been reported. Thus the extracellular pH has been determined by using molecular probes with a wavelength distribution dependent on pH, tumor oxygenation has been studied by imaging phosphorescence lifetime after administration of porphyrin probes, and the hemoglobin saturation has been determined by hyperspectral imaging [4, 13, 39]. Hyperspectral imaging requires a tunable wavelength filter, and this can be added to standard fluorescence microscopes to facilitate imaging of extracellular pH and hemoglobin saturation [13, 39]. However imaging of phosphorescence lifetime requires a specialized phosphorescence microscope and cannot be performed with the intravital microscopy assay reported here [4].

Our in-house-made window chamber was compatible with MRI. We compared *K*^trans^ images acquired by DCE-MRI with intravital microscopy images, and found that *K*^trans^ images reflected vascular morphology and function. This was an expected finding because *K*^trans^ mainly reflects blood perfusion in tumors with high vessel permeability [19]. We have previously shown that A-07 tumors have high permeability for macromolecules, and that *K*^trans^ reflects blood perfusion in this melanoma model [40, 41]. The MR-compatible window chamber can also be used to validate novel MR-techniques designed to provide information on tumor vasculature such as vessel size imaging and vessel architectural imaging [42, 43]. Moreover, other MR images such as diffusion weighted images and/or spectroscopy images may be combined with intravital microscopy images to provide complimentary information in comprehensive studies of the tumor microenvironment.

## Conclusion

An intravital microscopy assay that can be performed with a standard fluorescence microscope was presented, and this assay represents a useful and affordable alternative to intravital microscopy assays using confocal and multi-photon microscopes. The assay allowed quantification of morphological and functional parameters of normal tissue and tumor vasculature, and was used to study tumor growth and vascularization, tumor-associated lymphatics, and vascular effects of acute cyclic hypoxia and antiangiogenic treatment. Acute cyclic hypoxia induced angiogenisis resulting in increased densities of small-diameter vessels and increased BST values, whereas sunitinib treatment selectively removed small-diameter vessels, reduced BST values, and induced tumor hypoxia. The MR-compatible window chamber preparation enabled MRI and intravital microscopy of the same tumor, and DCE-MRI-derived *K*^trans^ images were shown to reflect vascular morphology and function.

## Supplementary Information

Online Resource 1 First-pass imaging movie of a representative R-18-GFP melanoma xenograft illustrating how a 0.2 ml bolus of 155 kDa TRITC-dextran moves through the vascular network of the tumor. The movie was recorded with a temporal resolution of 44.8 ms and a pixel size of 7.5 × 7.5 μm^2^. Single frames from the movie and relative signal intensity versus time curves for selected vessels are shown in Fig. 4a, and the blood supply time (BST) image and the BST histogram of the tumor are shown in Fig. 4b. (MP4 12.3 MB)
